# Flexible Solar-Blind Ultraviolet Photodetector Based on β-Ga_2_O_3_ Nanowire Channel Bridge Structure: Combining High Responsivity and Strain Stability

**DOI:** 10.3390/s25051563

**Published:** 2025-03-04

**Authors:** Jinyun Liu, Tengfei Ma, Huihui Tian, Wuxu Zhang, Zhaopeng Liu, Zhiyi Gao, Baoru Bian, Yuanzhao Wu, Yiwei Liu, Jie Shang, Run-Wei Li

**Affiliations:** 1 CAS Key Laboratory of Magnetic Materials and Devices, Ningbo Institute of Materials Technology and Engineering, Chinese Academy of Sciences, Ningbo 315201, China; 2 Zhejiang Province Key Laboratory of Magnetic Materials and Application Technology, Ningbo Institute of Materials Technology and Engineering, Chinese Academy of Sciences, Ningbo 315201, China; 3 Center of Materials Science and Optoelectronics Engineering, University of Chinese Academy of Sciences, Beijing 100049, China

**Keywords:** flexibility, solar-blind ultraviolet photodetector, flame detection, fire rescue, robotic skin

## Abstract

Solar-blind ultraviolet photodetectors are gaining attention for their high signal-to-noise ratio and strong anti-interference capabilities. With the rising demand for applications in high-strain environments, such as fire rescue robots and smart firefighting suits, a flexible photodetector that maintains stable performance under bending strain is needed. Current devices struggle to balance strain cycle stability and responsivity. This paper presents a β-Ga_2_O_3_ nanowire photodetector on a flexible ultra-thin silicon substrate, fabricated using microchannel engraving and chemical vapor deposition. The device achieves a responsivity of 266 mA W^−1^ without strain, with less than 5.5% variation in photogenerated current under bending strain (0–60°), and a response time of 360 ms. After 500 cycles of 60° bending, the photogenerated current changes by only 1.5%, demonstrating excellent stability and responsivity, with broad application potential in flame detection and biological sensing.

## 1. Introduction

Solar-blind ultraviolet photodetectors have gained widespread use due to their exceptional high signal-to-noise ratio and strong anti-interference capabilities, finding applications in areas such as flame detection, remote control, missile warning systems, and chemical/biological analysis [1,2,3,4,5,6]. Among these applications, flame detection plays a crucial role in industrial production, fire safety, and environmental monitoring. Since the 200–280 nm ultraviolet wavelength range in sunlight is completely absorbed by the ozone layer and does not reach the Earth’s surface, solar-blind ultraviolet photodetectors rely primarily on ultraviolet radiation emitted by flames in this range for detection. The light signals in this wavelength range are nearly free from background noise, offering a high signal-to-noise ratio and being less affected by lighting conditions, which makes them more adaptable and stable, thus making solar-blind ultraviolet photodetectors the dominant flame detection method [4,7]. With the increasing demand for complex application scenarios, such as fire rescue robots and smart firefighting suits, the development of a flexible solar-blind ultraviolet photodetector that can maintain stable performance under bending strain has become an urgent technical challenge.

Currently, solar-blind ultraviolet photodetectors predominantly rely on wide-bandgap semiconductor materials, such as AlGaN [8], ZnMgO [9], diamond [10], and Ga_2_O_3_ [11]. Among these, Ga_2_O_3_ is widely regarded as an excellent material for solar-blind ultraviolet photodetectors due to its direct bandgap (4.4–5.2 eV), which allows detection of the solar-blind spectrum without alloying, as well as its good thermal stability and high ultraviolet absorption [12]. Ga_2_O_3_ exists in five polymorphs, with the β-phase being the most stable solid phase; other metastable phases can transform into the stable β-phase under specific temperature and humidity conditions [13,14,15,16]. The optical bandgap of β-Ga_2_O_3_ is approximately 4.9 eV, with the absorption edge around 254 nm, and it exhibits high transmittance for near-ultraviolet and visible light [17]. Additionally, β-Ga_2_O_3_ displays characteristic emission peaks in the 290–365 nm, 390–450 nm, and 470–490 nm ranges [18]. β-Ga_2_O_3_ is available in bulk, film, and nanomaterial forms [11,12,19]. Compared to bulk and thin-film materials, β-Ga_2_O_3_ nanowires exhibit more significant surface effects and higher surface area, which increases surface energy and enhances the absorption efficiency of incident light due to internal reflection between nanowires [20,21,22], thereby leading to higher responsivity in solar-blind ultraviolet detection.

The preparation methods for β-Ga_2_O_3_ nanowires mainly include thermal oxidation [23], chemical vapor deposition (CVD) [13,24,25,26,27,28,29], radio frequency magnetron sputtering [30], molecular beam epitaxy [31,32], laser ablation [33], carbothermic reduction [34], and electrospinning high-temperature phase transition methods [35]. CVD-grown β-Ga_2_O_3_ nanowires exhibit high crystallinity and controlled growth conditions. However, the use of noble metal nanoparticles as catalysts may increase the substrate conductivity, thereby affecting the performance of the device. To reduce the impact of catalyst particles on device performance, Ding et al. [26] proposed engraving microchannels on the substrate, which allows the β-Ga_2_O_3_ nanowires to bridge across the microchannel, reducing dark current and improving responsivity.

In terms of flexible substrates, their application is crucial for the flexibility of the device. Currently, research mainly focuses on amorphous and β-Ga_2_O_3_ phases based on one-dimensional nanomaterials or thin films, fabricated on flexible substrates such as PET [36], PEN [37], ultra-thin mica [38], and fiberglass fabrics [39] to enhance the strain stability and robustness of the device. However, the preparation of β-Ga_2_O_3_ nanowires typically requires high growth temperatures (≥800 °C), which are much higher than the failure temperatures of traditional flexible substrates [37]. This makes it difficult for devices based on traditional flexible substrates to simultaneously achieve excellent optoelectronic conversion performance and good strain stability. Some flexible substrates with better high-temperature resistance, such as fiberglass fabrics [39], can balance strain capabilities and good optoelectronic performance, but their compatibility with traditional thin-film electrode preparation methods still needs further improvement. Therefore, the development of a flexible solar-blind ultraviolet photodetector with high responsivity under no strain and stable performance under strain conditions remains one of the urgent technical challenges to be addressed.

To address the low responsivity caused by the Au nanoparticle-catalyzed CVD method and the poor strain-cycle stability of high-temperature flexible substrate photodetectors, this paper proposes a solution using a thinned traditional silicon-based substrate as the flexible substrate combined with microchannel etching. This substrate not only has a certain degree of flexibility but also maintains stable physicochemical properties at high temperatures. The high-quality crystallized β-Ga_2_O_3_ nanowires grown by the CVD method do not damage the substrate’s flexibility, and the substrate is compatible with micro-nano processing and electrode fabrication, facilitating the preparation of flexible solar-blind ultraviolet detectors. At the same time, the method of etching microchannels through the growth substrate covered with catalyst particles is employed, enabling the grown β-Ga_2_O_3_ nanowires to bridge above the microchannels, forming a bridge-like structure. This microchannel structure effectively reduces the substrate conductivity, improves the dark current characteristics of the device, and enhances its responsivity in solar-blind ultraviolet detection. Therefore, the above solution is expected to improve the stability and reliability of the device in practical applications.

In this work, chemical vapor deposition (CVD) is employed to grow β-Ga_2_O_3_ nanowires with uniform morphology and high ultraviolet absorption on a flexible ultra-thin silicon substrate using Au nanoparticles as the catalyst. The effect of growth temperature and time on the nanowire morphology and ultraviolet absorption is studied. By engraving microchannels on the substrate, the β-Ga_2_O_3_ nanowires successfully bridge across the microchannel, effectively reducing the impact of catalyst particles on device performance. Finally, using microfabrication and electron beam deposition, an MSM (Metal–Semiconductor–Metal) type flexible solar-blind ultraviolet photodetector with high responsivity under no strain and stable performance under bending strain is constructed. The experimental results show that the device achieves a responsivity of 266 mA W^−1^ under no strain, and after 500 cycles of 60° bending strain, the photogenerated current changes by only 1.5%, indicating that the device maintains excellent detection performance under bending strain.

## 2. Materials and Methods

### 2.1. Materials

The ultra-thin silicon wafers used in the experiments were purchased from Shenzhen Silicon Crystal Electronics Technology Co., Ltd., Shenzhen, China, and the double-sided oxidized 500 nm SiO_2_ wafers were purchased from Hefei Yuanjing Technology Materials Co., Ltd., Hefei, China. High-purity Ga metal (99.99%) was purchased from Shanghai Maclin Biochemical Technology Co., Ltd., Shanghai, China.

### 2.2. Preparation of Ga_2_O_3_ Nanowires on Ultra-Thin Silicon Substrates

In this work, chemical vapor deposition (CVD) is employed, with gallium as the diffusion source, Au nanoparticles as the catalyst, and oxygen involved in the reaction, to grow Ga_2_O_3_ nanowires on the surface of an ultrathin silicon substrate. The tube furnace provided the required temperature and gas flow. The process steps are as follows:

Preparation of Au Nanoparticles on Ultra-Thin Silicon Substrates: Au nanoparticles were deposited as the catalyst for Ga_2_O_3_ nanowire growth using a JGP450 magnetron sputtering deposition system on an ultra-thin silicon wafer (thickness: 50 μm, size: 10 × 30 mm). The deposition conditions were as follows: Ar gas flow rate of 303 sccm, argon pressure of 3.3 Pa, and RF power of 20 W, with a sputtering time of 55–65 s to achieve an Au film thickness of approximately 10 nm. After deposition, rapid annealing was carried out in a vacuum high-temperature tube furnace (OTF-1200X, Hefei Kejing Materials Technology Co., Ltd., Hefei, China) at 900 °C. After annealing, the SiO_2_ substrate was cleaned, and liquid Ga metal was applied as the evaporation source.

CVD Growth of Ga_2_O_3_ Nanowires: This process includes mechanical etching of the growth substrate, placing the substrate and evaporation source, vacuuming, heating under a protective atmosphere, oxidation reaction, and cooling. The mechanical etching was carried out using a probe on the Au nanoparticles deposited and annealed, creating a 20 μm wide through-channel. This channel formation ensures that the Ga_2_O_3_ nanowires form a bridge across it, reducing the impact of dark current on device performance. During the process, the ultra-thin silicon substrate with and without Au nanoparticles was placed downstream in the gas flow, and the evaporation source was placed in the center of the tube furnace. After the vacuum level reached 10^−5^ Pa, Ar gas was introduced for gas circulation cleaning, then the temperature was increased at a rate of 10 °C/min to the reaction temperature (700 °C, 800 °C, or 900 °C). During the reaction, O_2_ (50 sccm) was introduced, and the reaction was maintained for 10 min, 30 min, or 60 min. After the reaction, the O_2_ valve and furnace were closed, and the material was removed after cooling.

Electrode Preparation: The electrodes were prepared by electron beam evaporation of a 100 nm Au film. The electron beam evaporation process applied a certain pre-bending stress to enhance electrode adhesion and stability during strain cycling.

### 2.3. Characterization

The microstructure of the sputtered Au film and its structure after high-temperature annealing were characterized by scanning electron microscopy (SEM). The structure, morphology, solar-blind UV absorption, and optical bandgap of the β-Ga_2_O_3_ nanowires were analyzed by X-ray diffraction (BRUKER, Billerica, MA, USA), SEM equipped with energy-dispersive X-ray spectroscopy (EDS), and a Lambda 950 UV-Vis spectrophotometer (P.E. Co., Waltham, MA, USA). The optoelectronic performance of the solar-blind UV photodetector was tested using a custom-built optoelectronic testing system. This system includes a Keysight B1500A semiconductor parameter analyzer (Keysight, Santa Rosa, CA, USA), a 254 nm solar-blind UV light source, a UV light power meter, UVC probe, and a timer. The strain application system was based on a precision linear T-type slide module with a miniature handwheel. The strain cycling system consists of a miniature electric precision linear T-type slide, power supply, driver, and programmable controller.

## 3. Results and Discussion

**Figure 1:**
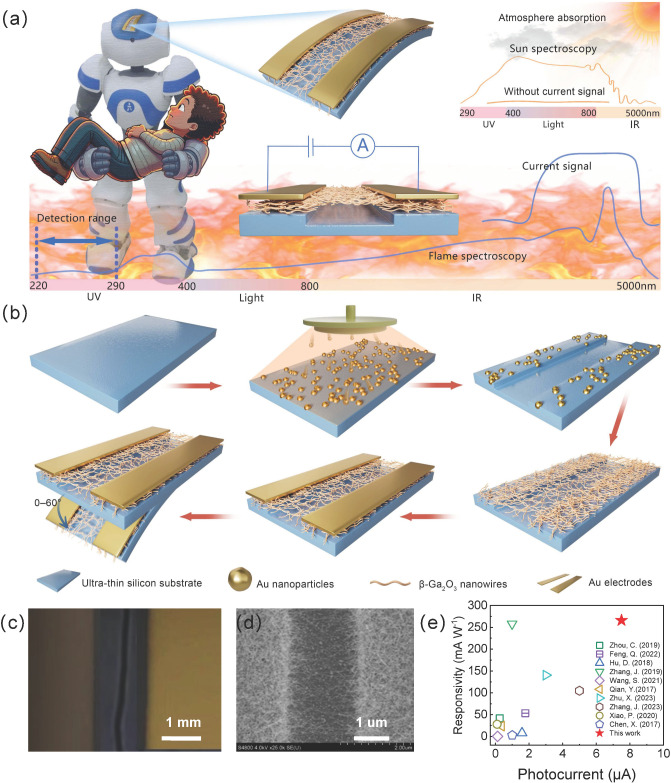
Design principle and fabrication process of flexible ultraviolet photodetector based on β-Ga_2_O_3_ nanowire trench-bridge structure. (**a**) The application of the photodetector in the working scenario of the fire rescue robot electronic skin and the flame signal detection mechanism. (**b**) Fabrication process of flexible ultraviolet photodetector based on β-Ga_2_O_3_ nanowire trench-bridge structure. (**c**) Optical microscope image of the flexible daylight-blind ultraviolet photodetector, and (**d**) SEM image. (**e**) Performance comparison of device responsivity and photocurrent of the flexible daylight-blind ultraviolet photodetector [9,17,36,40,41,42,43,44,45,46].

The design principle and application scenarios of the flexible solar-blind ultraviolet (UV) photodetector based on a β-Ga_2_O_3_ nanowire bridge structure are illustrated in Figure 1a. This detector is primarily employed in fire rescue robots’ electronic skin, enabling real-time sensing of flame intensity and converting it into electrical signals. In doing so, the robot can avoid high-intensity fire sources and promptly formulate optimal rescue paths for trapped personnel. By effectively eliminating the interference of solar radiation, the detector features high sensitivity, fast response, and flexibility, thereby enabling accurate detection of UV radiation in complex fire environments and adapting to various robot surfaces. Moreover, the high-temperature stability of β-Ga_2_O_3_ nanowires ensures reliable operation under extreme conditions, significantly enhancing the device’s reliability. Figure 1b depicts the fabrication process for the flexible β-Ga_2_O_3_ nanowire UV photodetector with the bridging structure. Detailed growth parameters are described in Section 2. The distribution of Au nanoparticles after high-temperature annealing is shown in Figure S1a, demonstrating a uniform layer of Au particles on the substrate, thereby offering ideal catalytic conditions for subsequent Ga_2_O_3_ nanowire growth.

First, we investigated how the catalyst influences the morphology of Ga_2_O_3_ grown by CVD. Under a growth temperature of 800 °C and a duration of 30 min, Ga_2_O_3_ was deposited on ultra-thin silicon substrates with and without Au nanoparticle catalysts, respectively. Figure S1b,c present the resulting microstructures, revealing that Ga_2_O_3_ nanowires exhibit one-dimensional (1D) structures with diameters of around 70–100 nm. Under catalyst-free conditions, the nanorods have an aspect ratio of less than 20, and each rod is relatively isolated. By contrast, when Au nanoparticles are used, the nanowires exhibit a noticeably larger aspect ratio and intertwine with one another, increasing the effective light absorption area. These interwoven nanowires not only facilitate efficient electron transport but also alleviate mechanical fracture under strain due to the interstitial space between nanowires.

### 3.1. Synthesis and Performance Analysis of β-Ga_2_O_3_ Nanowires on Ultra-Thin Silicon Substrates

#### 3.1.1. Effect of Growth Temperature and Time on the Morphology of Ga_2_O_3_ Nanowires Prepared by Au-Catalyzed CVD

High aspect ratio nanowires exhibit significant surface effects and a high specific surface area, with their surface area being much larger than their volume. This allows more electrons and photons to interact with their surface, thereby enhancing photon absorption efficiency and further improving optoelectronic conversion efficiency. Additionally, the internal reflection effect between high aspect ratio nanowires helps to increase the absorption of incident light [32]. Therefore, this study chooses high aspect ratio β-Ga_2_O_3_ nanowires as the preferred morphology. However, nanowires with large aspect ratios may also be associated with more surface defects and surface states, which can increase the recombination rate of charge carriers and negatively impact optoelectronic conversion efficiency. As a result, precisely optimizing surface characteristics to enhance optoelectronic performance remains a key direction for future research. By varying the growth temperature (700 °C, 800 °C, 900 °C) and growth duration (10 min, 30 min, 60 min), Ga_2_O_3_ nanowires were successfully synthesized on ultra-thin silicon substrates under various process conditions. Their microstructures were observed (see Figure S2). At 700 °C for 30 min, the material forms short, relatively thick 1D structures (Figure S2a), implying that lower temperature leads to a mixture of amorphous and crystalline phases, preventing complete crystallization and hindering the formation of nanowires with a high aspect ratio. At 800 °C for 30 min, the material appears as uniform nanowires with diameters of 50–60 nm and a high aspect ratio, while a small fraction of the nanowires grow laterally to form 2D nanostructures (Figure S2b). This observation indicates that elevated temperature promotes axial growth and improves morphological uniformity. At 900 °C for 30 min, the nanowires become thicker, forming nanopillars, and exhibit twisting or branching (Figure S2c). As temperature rises, higher vapor pressure can lead to supersaturation, prompting lateral growth without Au catalytic droplets at the tips. Such conditions may trigger re-nucleation on the nanowire surface, forming thicker nanowires and potential crystalline defects (e.g., impurity defects, stacking faults), thus reducing crystalline quality.

Shortening the duration at 800 °C to 10 min yields a relatively small quantity of thin, high-aspect-ratio nanowires (Figure S2d). Although these nanowires meet the desired morphology, their yield is low. This implies that 800 °C is conducive to Ga oxidation and Ga_2_O_3_ nucleation, favoring axial growth. However, the brief growth time is insufficient to produce large amounts of nanowires. When the growth is extended to 60 min at 800 °C, copious nanowires with large aspect ratios interlace with each other, accompanied by substantial 2D structures (e.g., nanosheets or “nanoflags”) (Figure S2e). Prolonged growth can cause the Au catalyst to evaporate or diffuse toward nanowire sidewalls and the substrate, leading to lateral nucleation of 2D nanoflags. Once the Au catalyst evaporates, extending the duration no longer increases the nanowire yield.

These observations indicate that 800 °C and 30 min provide the optimal conditions for promoting axial nanowire growth [47], thereby yielding nanowires with the desired morphology. In addition, we also observed that the nanowires exhibit a phenomenon of winding/knotting. Moderate winding helps to improve the stability of the device under cyclic bending stress. On the one hand, the winding structure effectively reduces the mechanical anisotropy of the nanowires in different directions, thereby maintaining the stability of the overall morphology. On the other hand, the winding structure helps to alleviate some of the stress generated by bending strain, enhancing the stability of the optoelectronic signal under bending conditions. The X-ray diffraction (XRD) pattern (Figure S2f) of nanowires prepared under these conditions (800 °C, 30 min) was compared with the standard data (JCPDS #43-1012). The distinct diffraction peaks corresponding to the (−201), (−401), (002), (−111), (111), (−402), (−601), and (403) planes confirm the monoclinic β-Ga_2_O_3_ phase. The strong and narrow diffraction peaks suggest good crystallinity. We further analyzed the growth mechanism of Ga_2_O_3_ nanowires synthesized by Au-catalyzed CVD on ultra-thin silicon substrates by investigating microstructural evolution over growth times of 0 min, 10 min, 20 min, and 30 min at 800 °C. Detailed analyses and results are provided in the supplementary materials [47,48] (Figure S3).

In summary, adjusting parameters such as the catalyst, growth time, and temperature in CVD, combined with structural characterizations, successfully yielded β-Ga_2_O_3_ nanowires with large aspect ratios, uniform morphology. Further characterization and theoretical analyses of the growth process verified the growth mechanism, establishing a robust material foundation for fabricating flexible solar-blind UV photodetectors.

#### 3.1.2. Effect of Growth Conditions on Solar-Blind UV Absorption and Optical Bandgap of Ga_2_O_3_ Prepared by Au-Catalyzed CVD

Five sets of Ga_2_O_3_ samples grown under different temperatures and durations were evaluated for their optical transmittance and absorption via a UV-Vis spectrometer (see Figure S4). Their bandgaps were estimated using Equation (1):

(1) $${\left(\alpha \mathrm{hv}\right)}^{2}=B\left(hv-E_{g}\right),$$

where *α* is the absorption coefficient, *h*ν is the photon energy, and *E*_g_ is the optical bandgap. By plotting (*αh*ν)^2^ versus *h*ν and extrapolating the linear region to intersect the energy axis, the bandgap can be estimated.

All samples show relatively strong absorption and low transmittance between 200 nm and 280 nm (solar-blind UV range). The absorption edge of the samples grown at 800 °C or 900 °C is around 260 nm, whereas absorption in the UV-Visible range is negligible, illustrating excellent solar-blind UV selectivity. Conversely, the absorption edge red-shifts to ~290 nm for samples grown at 700 °C for 30 min, making them unsuitable for solar-blind UV detection due to diminished selectivity. The estimated bandgaps of the CVD-grown samples are compiled in Table S1. Samples grown at 800 °C, 30 min; 800 °C, 60 min; and 900 °C, 30 min exhibit larger bandgaps and higher solar-blind UV absorption. However, samples at 800 °C, 60 min and 900 °C, 30 min manifest non-uniform morphologies (e.g., 2D nanosheets and nanoflags), potentially compromising the strain stability of flexible devices. By contrast, 800 °C and 30 min provides a better trade-off, with desirable optoelectronic performance, reduced fabrication cost, and higher efficiency, thus well-suited for solar-blind UV photodetection.

### 3.2. Fabrication of the β-Ga_2_O_3_ Nanowire Flexible Solar-Blind UV Detector

The MSM-structured β-Ga_2_O_3_ nanowire solar-blind UV photodetector offers simple processing, low leakage current and junction capacitance, and strong compatibility with various fabrication procedures–features well-suited for flexible devices. Such a photodetector allows investigations of optoelectronic performance under bending strain to achieve strain-stable solar-blind UV photodetection. Using Au nanoparticles as catalysts, β-Ga_2_O_3_ nanowires were grown at 800 °C for 30 min and integrated into an MSM configuration for a flexible solar-blind UV detector. Before growth, a microchannel ~20 µm wide was etched onto an ultra-thin silicon substrate. Consequently, only a pair of Au electrodes needed to be deposited on opposite sides of the channel (Figure 1b). A Schottky junction is formed between the Au electrodes (work function 5.1 eV) and the β-Ga_2_O_3_ nanowires (work function 4.00 ± 0.05 eV). Detailed electrode fabrication steps are given in Section 2.

Figure 1c,d present the optical micrograph and SEM images of the fabricated flexible solar-blind UV photodetector. A ~20 µm wide microchannel is clearly visible, bridged by numerous high-aspect-ratio β-Ga_2_O_3_ nanowires that intertwine above the channel, forming the designed bridging architecture. Hence, an MSM-type flexible solar-blind UV photodetector was successfully realized by patterning Au electrodes on either side of a microchannel on the ultra-thin silicon substrate. Subsequent optoelectronic performance characterization and strain stability tests were conducted based on this device.

To evaluate the responsivity and photocurrent levels of this device, we compare previously reported photocurrent and responsivity data on flexible solar-blind UV photodetectors made from β-Ga_2_O_3_ 1D nanomaterials, α-Ga_2_O_3_ 1D nanomaterials, and Ga_2_O_3_ thin films, shown in Figure 1e [9,17,36,40,41,42,43,44,45,46]. Our detector exhibits a relatively high responsivity in the unstrained state, underscoring its excellent performance.

### 3.3. Optoelectronic Performance of the Flexible β-Ga_2_O_3_ Nanowire Solar-Blind UV Detector

#### 3.3.1. Current–Voltage (I–V) Characteristics

**Figure 2:**
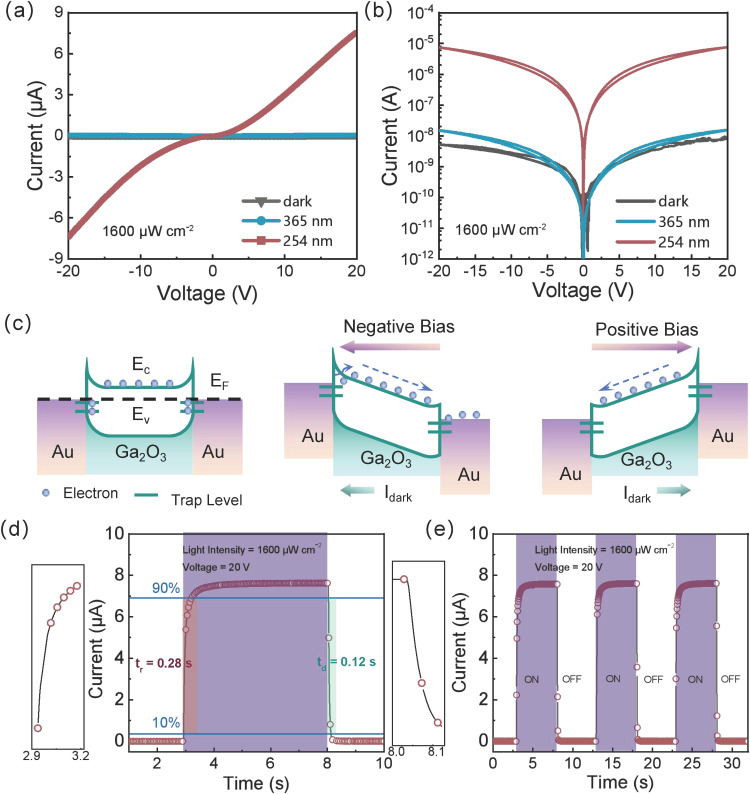
Photoelectric performance of flexible daylight-blind ultraviolet photodetector based on β-Ga_2_O_3_ nanowires on ultrathin silicon substrate. The I–V characteristic curves of the detector under dark, 254 nm, and 365 nm illumination in (**a**) linear scale and (**b**) logarithmic scale. (**c**) Schematic energy band diagrams of the device under different biases: (**left**) no bias, (**middle**) negative bias, and (**right**) positive bias. (**d**) I–T characteristic curve for a single cycle under 254 nm illumination on/off and (**e**) I–T characteristic cycle curve for three cycles.

The I–V characteristic is a critical metric for solar-blind UV photodetectors. The schematic diagram and actual setup of our optoelectronic measurement system are detailed in Figure S5. Under dark conditions and irradiation at 254 nm and 365 nm with a power density of 1600 µW cm^−2^, the I–V curves of the detector were measured from −20 V to +20 V, as shown in Figure 2a,b. At +20 V, the dark current is 7.91 × 10^−9^ A, and at −20 V, it is 5.18 × 10^−9^ A, indicating an extremely high resistance and minimal defects. By bridging high-aspect-ratio nanowires across the microchannel, the dark current is effectively reduced. Under 365 nm illumination, the photocurrent is 5.49 × 10^−8^ A at +20 V, and 5.34 × 10^−8^ A at −20 V. By contrast, at 254 nm (solar-blind UV), the photocurrent is 2.01 × 10^−10^ A at 0 V, and 7.58 × 10^−6^ A at +20 V (7.43 × 10^−6^ A at −20 V).

Thus, the detector responds weakly to 365 nm due to insufficient photon energy to excite carriers across the bandgap, while showing a marked photocurrent under 254 nm illumination. The dual Schottky barriers in the device also help reduce dark current. In Figure 2b, the minimal dark current offset and asymmetry mainly arise from differences in work functions, leading to Schottky barriers and trap states that capture and release electrons, causing the captured current to be lower than the released current. The more oxygen vacancy defects in the material, the more trap states it has; hence, the more pronounced the current offset [42].

This non-symmetric dark current in the I–V curves can be interpreted via the capture-release mechanism of trap states. As Ga_2_O_3_ has a higher Fermi level than Au [49,50], electrons diffuse from Ga_2_O_3_ to Au, forming two Schottky barriers. Electrons occupying trap states below the Fermi level are depicted in Figure 2c (left) [42]. Under large reverse bias (Figure 2c (middle)), electrons in trap states are released, substantially increasing the reverse dark current, making it exceed the forward dark current. Conversely, at large forward bias (Figure 2c (right)), fewer electrons are released from trap states, resulting in lower forward dark current. Under 254 nm illumination, fewer interfacial trap states remain, weakening the capture-release effect, which makes the device’s I–V curve more symmetric in the illuminated condition.

#### 3.3.2. Response and Recovery Time

Response time, or rise time, is defined as the duration for the current to increase from 10% to 90% of its peak value upon switching on the light at a given bias voltage. Recovery time, or fall time, is the duration for the current to decrease from 90% to 10% of its peak value when the light is switched off [37]. The time-resolved I–T curves thus provide a means to quantify the device’s response speed and stability. Here, a millisecond timer controlled the on/off cycles (5 s per cycle) of a 254 nm solar-blind UV lamp (intensity of 1600 µW cm^−2^), at a sampling frequency of 25 Hz and a bias of +20 V (Figure 2d,e). From Figure 2d, the detector’s response and recovery times are about 283 ms and 124 ms, respectively, indicating a fast response to solar-blind UV. The current plateau at the peak indicates that the UV illumination duration is sufficient for the current to reach equilibrium. Figure 2e shows three successive on/off cycles, revealing a highly reproducible response: The current consistently returns to its initial dark level after the light is turned off. Each cycle’s rise and fall times, as well as the peak photocurrent, are nearly identical, underscoring the stability and reproducibility of the device’s photoresponse.

Reported solar-blind UV photodetector response times span microseconds [37] to seconds [42], primarily governed by the rate at which carrier concentrations change. Oxygen vacancies in Ga_2_O_3_ act as carrier traps or recombination centers, often prolonging response times. For undoped β-Ga_2_O_3_ nanowires on a silicon substrate, controlling the oxygen content can adjust the concentration of oxygen vacancies to optimize device response speed. However, response speed and responsivity are often in conflict, making it challenging to achieve both simultaneously. Still, a millisecond-scale response is sufficient for most applications, including flame detection.

#### 3.3.3. Influence of Optical Power Density

**Figure 3:**
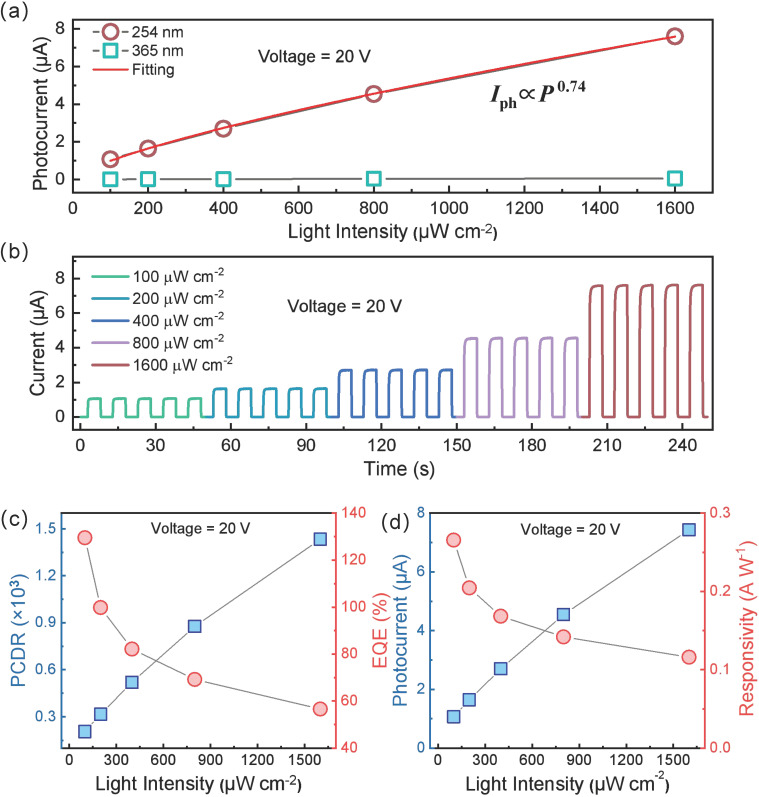
The effect of optical power density on the photoelectric performance of the flexible daylight-blind ultraviolet photodetector based on β-Ga_2_O_3_ nanowires on ultrathin silicon substrate: (**a**) Relationship between optical power density and photocurrent. (**b**) I–T cycle response curves at different optical power densities. (**c**) Variation in PCDR and ECQ with optical power density. (**d**) Variation in photocurrent and responsivity (R) with optical power density.

As optical power density rises, more electron–hole pairs are generated, increasing the photocurrent. Figure 3a shows the correlation between power density and photocurrent, fitting to a sublinear relation *I*_ph_ = CP^α^, α = 0.74, α < 1. This sublinearity arises from oxygen-vacancy defects that serve as trap states or recombination centers. Under higher power illumination, additional carriers are trapped or undergo accelerated recombination, weakening the linear correlation [51].

The periodic response at +20 V bias under 254 nm solar-blind UV light with power densities from 100 µW cm^−2^ to 1600 µW cm^−2^ is shown in Figure 3b. At 1600 µW cm^−2^, the peak photocurrent reaches 7.43 µA, whereas a lower intensity of 100 µW cm^−2^ still yields 1.07 µA, indicating that the device can detect low-intensity solar-blind UV. For each power level, the rise and fall times remain consistent across five cycles, and the device’s photocurrent quickly stabilizes at each peak. These consistent, distinct responses under varying optical powers show the device’s feasibility for array-based imaging applications.

The photo-to-dark current ratio (PCDR) is another key figure of merit, defined by Equation (2). From the I–V data in Figure 2a,b, the PCDR under 20 V bias and 254 nm/365 nm illumination (1600 µW cm^−2^) is 958 and 6.9, respectively; at −20 V, the PCDR is 1434 and 10.3. Thus, the PCDR for 254 nm is nearly 100 times greater than for 365 nm, confirming the device’s selectivity toward solar-blind UV. Figure 3c shows that PCDR under 254 nm increases nonlinearly as power density increases (100–1600 µW cm^−2^). Since photocurrent is approximately three orders of magnitude higher than dark current, PCDR primarily reflects changes in photocurrent. Consequently, PCDR and photocurrent follow a similar trend.

(2) $$\mathrm{PCDR}=\frac{I_{\lambda }-I_{dark}}{I_{dark}}$$

*I*_λ_ is the response current with a wavelength of λ, and *I*_dark_ is the dark current in a dark environment.

External quantum efficiency (EQE), which represents the ratio of collected electrons to incident photons, is given by Equation (3). At a fixed wavelength, EQE is linearly related to responsivity (R). As shown in Figure 3c, EQE decreases gradually with increasing power density, slowing its rate of decline over time. Under 254 nm irradiation at 100 µW cm^−2^, the EQE reaches 129%, exceeding 100% due to possible secondary absorption processes. When the power density is 200 µW cm^−2^, the EQE drops to 99.8% and further down to 57% at 1600 µW cm^−2^. This decrease can be attributed to trap states and recombination centers introduced by defects, which capture photogenerated carriers or accelerate their recombination.

(3) $$\mathrm{ECQ}=\frac{hc\times R_{\lambda }}{e\times \lambda }\times 100\%$$

where *h* is Planck’s constant, c is the speed of light, e is the elementary charge, *λ* is the incident wavelength, and *R*_λ_ is the responsivity at that wavelength *λ*.

Responsivity (*R*) characterizes the ratio of photocurrent to incident optical power, typically in A W^−1^, as expressed by Equation (4). Experimentally, R decreases with increasing optical power from 100 µW cm^−2^ to 1600 µW cm^−2^, as shown in Figure 3d. At a power density of 100 µW cm^−2^, the device’s maximum responsivity to 254 nm is 0.266 A W^−1^, yielding a photocurrent of 1.07 × 10^−6^ A. Defects, such as oxygen vacancies, cause non-linear growth in photocurrent, implying that the photocurrent’s increase is slower than that of power density. As a result, the rate of increase in photocurrent is lower than the rate of increase in light power density, causing the device’s responsivity to gradually decrease as the incident light power increases.

(4) $$R=\frac{I_{\lambda }-I_{dark}}{P\times S}$$

where *P* is the incident power and *S* is the effective photosensitive area.

Specific detectivity (D*) is a crucial figure of merit for weak-signal detection, normalizing the responsivity (*R*), photosensitive area (*S*), and dark current, as shown in Equation (5) [44]:

(5) $$D^{*}=\frac{\sqrt{S}\times R}{\sqrt{2qI_{dark}}}$$

where *q* is the elementary charge. At 100 µW cm^−2^, the detector’s D* is 1.30 × 10^12^ Jones, comparable to existing Ga_2_O_3_-based solar-blind UV photodetectors [44], underscoring its strong detection capabilities.

#### 3.3.4. Influence of Bias Voltage

**Figure 4:**
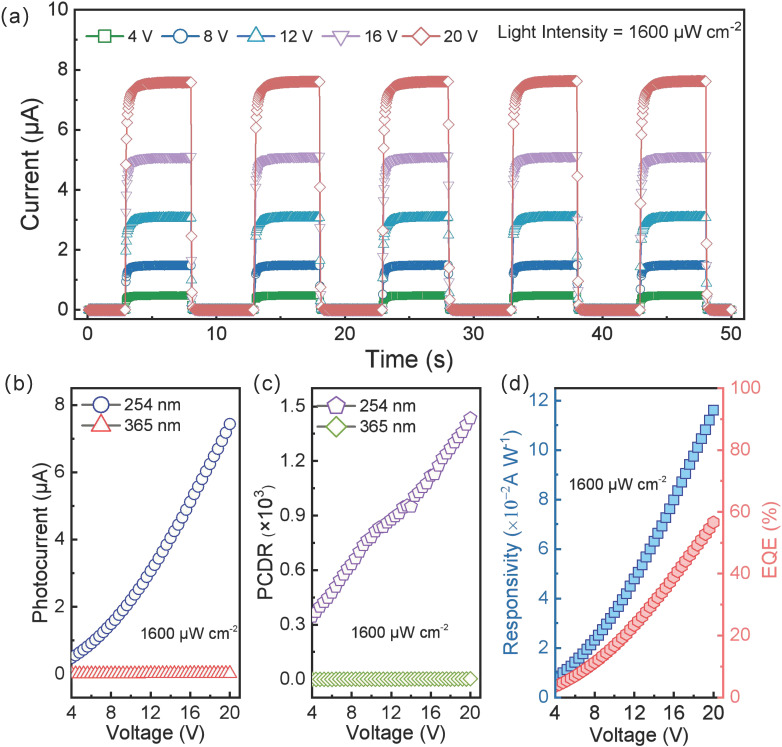
The effect of bias on the photoelectric performance of the flexible daylight-blind ultraviolet photodetector based on β-Ga_2_O_3_ nanowires: (**a**) I–T characteristic curves at different bias voltages under 254 nm wavelength. (**b**) Relationship between photocurrent and bias voltage at different wavelengths. (**c**) Relationship between PCDR and bias voltage at different wavelengths. (**d**) Relationship between responsivity (R) and external quantum efficiency (EQE) with bias voltage at 254 nm wavelength.

Applying a larger bias enhances the separation of photogenerated electron–hole pairs, thus increasing photocurrent but also affecting other device characteristics. Under 254 nm solar-blind UV irradiation (1600 µW cm^−2^), we tested the device’s I–T response under biases of 4 V, 8 V, 12 V, 16 V, and 20 V, as shown in Figure 4a. The peak photocurrent rises with increasing bias, while remaining stable at each bias level. Figure 4b plots the peak photocurrent under various voltages, rising from 0.48 µA at 4 V to 7.43 µA at 20 V. Higher bias facilitates carrier separation, thereby enhancing photocurrent. Meanwhile, the response to 365 nm UV barely changes, reaffirming the device’s solar-blind UV selectivity.

We further examined the effects of bias on PCDR, R, and EQE, shown in Figure 4c,d. As bias increases, PCDR significantly rises; for instance, PCDR is 344 at 4 V versus 1434 at 20 V, mainly because high bias substantially increases the photocurrent. Responsivity (*R*) and EQE similarly exhibit positive correlations with bias (Figure 4d). At 4 V, R is 0.007 A W^−1^ and EQE is 3.6%; at 20 V, *R* increases to 0.116 A W^−1^ and EQE to 56.6%. With constant *P* and *S*, the dark current is ~3 orders of magnitude smaller than the photocurrent. Therefore, raising the bias further boosts photocurrent, enhancing both responsivity and EQE.

In summary, the flexible β-Ga_2_O_3_ nanowire solar-blind UV detector on ultra-thin silicon exhibits excellent optoelectronic performance. Specifically, it features a low dark current (5.18 × 10^−9^ A at −20 V, 1600 µW cm^−2^) and a high solar-blind photocurrent (7.58 × 10^−6^ A at +20 V, 1600 µW cm^−2^). The photocurrent shows sublinear growth with respect to incident power density, yet increases with bias. The stable and highly repeatable peak photocurrent, along with selective solar-blind UV detection, confirms the device’s reliability. Its response time is in the millisecond regime, about 283 ms for rise and 124 ms for decay (+20 V, 1600 µW cm^−2^). PCDR positively correlates with both power density and bias, reaching 1434 at 20 V and 1600 µW cm^−2^. Responsivity decreases with higher incident power but rises with bias, reaching a maximum of 0.266 A W^−1^ at 20 V and 100 µW cm^−2^. EQE aligns with responsivity, peaking at 129% under 254 nm irradiation (20 V, 100 µW cm^−2^). Finally, the device’s maximum detectivity D^*^ is 1.30 × 10^12^ Jones (20 V, 100 µW cm^−2^). These strong unstrained-state results lay the groundwork for exploring the effects of bending strain.

### 3.4. Strain Stability Analysis of the Flexible β-Ga_2_O_3_ Nanowire Solar-Blind UV Detector

#### 3.4.1. Device Performance Under Bending Strain

To enhance strain stability of the ultra-thin silicon substrate, an additional flexible backing is applied on the rear side of the completed β-Ga_2_O_3_ nanowire detector. When the ultra-thin silicon is attached to a flexible substrate, the stress can be estimated using the Stoney equation [52,53,54]:

(6) $$\sigma_{f}=\frac{{\left(E_{s}t_{s}\right)}^{2}}{6\left(1-\Theta \right)t_{f}R}$$

where *t*_s_ and *t*_f_ are the thicknesses of the flexible substrate and the ultra-thin silicon, respectively, and Θ is Poisson’s ratio.

The stress on the ultra-thin silicon is proportional to the flexible substrate’s Young’s modulus and the square of the flexible substrate thickness. Consequently, selecting a low-modulus substrate (e.g., PDMS with 1–2 MPa, lower than TPU ~97 MPa or PET > 1000 MPa) and using a thin silicon layer help reduce stress from bending [52,53,55,56]. Hence, we coat a layer of PDMS (mixed at 1:10 ratio) on the backside of the ultra-thin silicon substrate to improve strain stability. The strain application and notation are shown in Figure S6, and details of the strain application and cycling system are provided in Figure S7.

**Figure 5:**
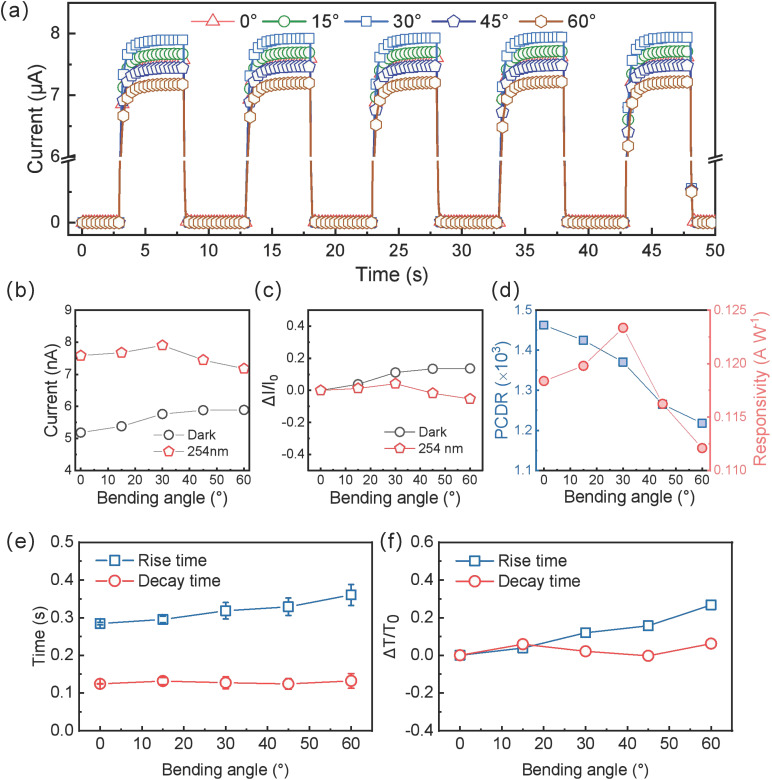
Bending strain stability of flexible daylight-blind ultraviolet photodetector based on β-Ga_2_O_3_ nanowires. (**a**) I–T characteristic curves under different bending states. (**b**) Dark and photocurrent under different bending states. (**c**) Variation rate of dark and photocurrent. (**d**) PCDR and responsivity (R) under different bending states. (**e**) Response time and recovery time under different bending states. (**f**) Variation rate of response time and recovery time.

At bending angles of 0°, 15°, 30°, 45°, and 60°, the device was tested under 254 nm solar-blind UV light with intensity 1600 µW cm^−2^ and a bias of +20 V, performing time-resolved measurements shown in Figure 5a. From 0° to 30° bending, the device’s photocurrent slightly increases, then decreases from 30° to 60°. Possible explanations include the following: minor variations in the contact between device electrodes and probes, leading to negligible measurement errors that reduce photocurrent; slightly larger photosensitive area and smaller distance to the UV source when the device is bent, marginally raising light intensity on the detector; beyond a certain strain threshold, some nanowire pathways may break, lowering photocurrent. In five consecutive measurement cycles at each bending angle, the peak photocurrent remains reproducible, revealing stable I–T curves under strain.

Figure 5b compares the dark current and photocurrent at various bending angles. The dark current fluctuates in single measurements but remains in the range of 5.18–5.89 nA across multiple tests. The photocurrent first rises then falls with increasing strain, reaching its maximum (7.90 µA) at 30° and minimum (7.18 µA) at 60°, reflecting the interplay of measurement errors, photosensitive area changes, light intensity variations, and partial nanowire breakage. Figure 5c shows how the dark and photocurrent change rates evolve with bending angle. Dark current gradually increases with angle, reaching a 13.7% rise at 60° compared to 0°. Photocurrent variation follows an initial rise and subsequent decline: From 0° to 30°, it increases by ~4.2% but decreases to −5.3% at 60°. Overall, the photocurrent and dark current remain relatively stable, with the photocurrent variation within ±5.5%.

Consequently, we quantify the photo-to-dark current ratio (PCDR) and responsivity (*R*) at each bending angle. As seen in Figure 5c, PCDR decreases slightly from 1462 at 0° to 1217 at 60°, still remaining in the 10^3^ range. For bending up to 30°, dark current grows faster than photocurrent; beyond 30°, the dark current continues to rise while the photocurrent slightly declines, leading to a slight drop in PCDR. Meanwhile, the device’s responsivity first increases then decreases with increasing angle, peaking at 0.123 A W^−1^ at 30°, then dropping to 0.112 A W^−1^ at 60°. Because the difference between photocurrent and dark current is large, the final trend in R mainly depends on photocurrent changes. Even at 60°, both PCDR and R vary by only about 10%, revealing good strain stability.

Variation in response and recovery time under bending is also critical for assessing strain stability. Figure 5e,f show that recovery time remains almost unchanged (0.12–0.13 s), with small error bars, indicating excellent reproducibility. By contrast, the response time increases from 0.28 s to 0.36 s as bending exceeds 30°, and the error bars become more pronounced—likely due to partial nanowire breakage or detachment from electrodes, impairing carrier transport. Figure 5f shows that the variation in recovery time stays within −0.2% to 6.0%, whereas the response time variation gradually rises from 4% up to 26.7% at 60°.

In summary, although bending strain increases the response time up to 360 ms (~26.7% change), the detector’s recovery time remains stable (within 6%). The total change in response speed is ~22.5%, yet it is still in the millisecond range, suitable for most solar-blind UV detection scenarios involving bending.

#### 3.4.2. Device Performance After Strain Cycling

**Figure 6:**
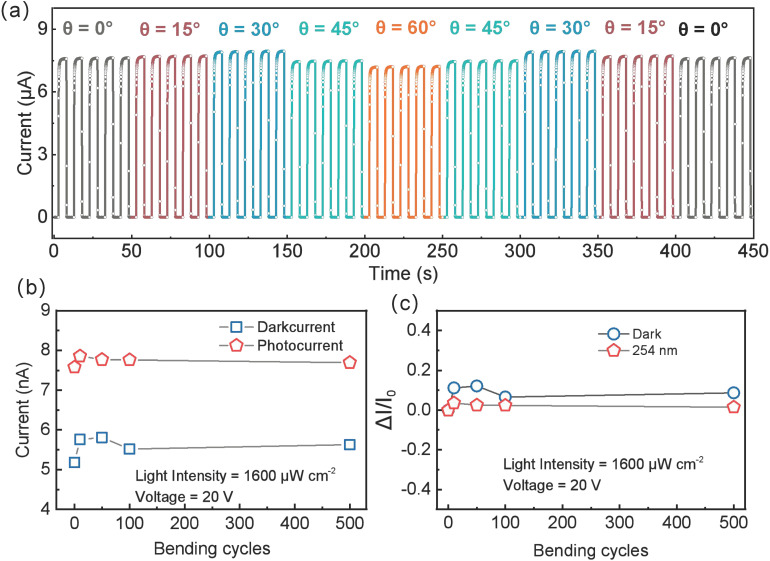
Performance analysis of flexible daylight-blind ultraviolet photodetector based on β-Ga_2_O_3_ nanowires after strain cycling. (**a**) Single cycle I–T curve under bending from 0° to 60° and back to 0°. (**b**) Variation in photocurrent and dark current with the number of bending cycles in the range of 0° to 60°. (**c**) Variation rate of photocurrent and dark current with the number of bending cycles.

Under 254 nm solar-blind UV illumination at 1600 µW cm^−2^, the device was subjected to bending cycles from 0° to 60° and back to 0°, with five consecutive I–T measurements at 20 V bias recorded for each angle in a single cycle. As shown in Figure 6a, the peak photocurrent remains nearly constant within each single cycle, and each bending angle exhibits consistent measurements. The stress application process (0° → 60°) and release process (60° → 0°) exhibit symmetrical I–T trends, suggesting no irreversible damage within a single cycle. The peak photocurrent is highest at ~30° and lowest at 60°, but the difference is small overall, implying stable performance under single-cycle bending.

To investigate multi-cycle strain effects, the device was subjected to 0–60° bending for 0, 10, 50, 100, and 500 cycles. After these cycles, the photocurrent and dark current at flat (0°) state were measured multiple times and averaged. As shown in Figure 6b,c, both photocurrent and dark current initially rise before falling and eventually stabilizing with increasing cycle count. The photocurrent peaks at 7.85 µA after 10 cycles, then declines slightly to 7.69 µA after 500 cycles. The dark current is minimal (5.18 nA) before strain cycling, reaches 5.81 nA after 50 cycles, then shifts slightly to 5.63 nA at 500 cycles. Figure 6c indicates that these variations remain modest. The maximal photocurrent shift is 3.6% at 10 cycles; it changes by only 1.5% after 500 cycles. The dark current fluctuates more but still remains within ±12.2%. In addition to measurement errors, minor degradation in electrode–nanowire contact may cause slight performance fluctuations.

Hence, even after 500 bending cycles at 60°, the device retains stable photocurrent and dark current, confirming its excellent stability and reliability under repeated strain.

## 4. Conclusions

This study presents a flexible solar-blind UV photodetector based on β-Ga_2_O_3_ nanowires with a bridge structure, utilizing an ultra-thin silicon substrate and Au nanoparticle catalysts. The device, fabricated via chemical vapor deposition (CVD), demonstrated excellent performance with a low dark current of 5.18 nA, high photocurrent of 7.58 µA, fast response times, and strong selectivity for the solar-blind UV range. It also showed a peak responsivity of 0.266 A/W, a maximum photo-to-dark current ratio of 1434, and an external quantum efficiency (EQE) of 129%. Importantly, the device maintained stable performance under bending strain, with minimal photocurrent variation (5.5%) up to 60° and only a 1.5% change after 500 bending cycles, demonstrating excellent mechanical flexibility and strain stability. The ultra-thin silicon substrate ensured high temperature and chemical stability, supporting sustained high responsivity even under strain. These results highlight the device’s potential for real-world applications, such as flame detection and fire rescue robotics, while offering a robust platform for flexible solar-blind UV photodetectors in dynamic environments.

## Data Availability

The data presented in this study are available on request from the corresponding author.

## Supplementary Materials

The following supporting information can be downloaded at https://www.mdpi.com/article/10.3390/s25051563/s1, Figure S1: Preparation of Au nanoparticles and the effect of the catalyst on the morphology of Ga_2_O_3_ nanowires grown by CVD; Figure S2: The effect of growth temperature and time on the morphology of Ga_2_O_3_ nanowires prepared by catalyst-assisted CVD method; Figure S3: Growth mechanism of Ga_2_O_3_ nanowires prepared by CVD method on ultrathin silicon substrates; Figure S4: The effect of different growth parameters on the daylight-blind ultraviolet absorption and optical bandgap of Ga_2_O_3_ nanowires prepared by catalyst-assisted CVD method; Figure S5: (a) Schematic diagram and (b) photograph of the optoelectronic testing system; Figure S6: Schematic diagram of the bending of β-Ga_2_O_3_ nanowire flexible daylight-blind ultraviolet photodetector; Figure S7: Strain application and strain cycling system; Table S1: Estimation results of the optical bandgap of Ga_2_O_3_ nanowires prepared by catalyst-assisted CVD method with different growth parameters.
