# Supplementary material for: Flexible Solar-Blind Ultraviolet Photodetector Based on β-Ga2O3 Nanowire Channel Bridge Structure: Combining High Responsivity and Strain Stability
Source: Sensors (Basel). 2025 Mar 4;25(5):1563. doi: 10.3390/s25051563 (PMC11902719; doi:10.3390/s25051563)
Supplement: Supplementary file 1 [file sensors-25-01563-s001.zip › sensors-3489304-supplementary.pdf]

Supporting Information

Flexible Solar-Blind Ultraviolet Photodetector Based on β-Ga_2O_3 Nanowire Channel Bridge Structure: Combining High Responsivity and Strain Stability

Jinyun Liu [1,2,3,†], Tengfei Ma [1,2,†], Huihui Tian [1,2,3,†], Wuxu Zhang [1,2], Zhaopeng Liu [1,2], Zhiyi Gao [1,2], Baoru Bian [1,2], Yuanzhao Wu [1,2], Yiwei Liu [1,2], Jie Shang [1,2,3,*] and Run-Wei Li [1,2,3,*]

[1] CAS Key Laboratory of Magnetic Materials and Devices, Ningbo Institute of Materials Technology and Engineering, Chinese Academy of Sciences, Ningbo 315201, China
2 Zhejiang Province Key Laboratory of Magnetic Materials and Application Technology, Ningbo Institute of Materials Technology and Engineering, Chinese Academy of Sciences, Ningbo 315201, China
[3] Center of Materials Science and Optoelectronics Engineering, University of Chinese Academy of Sciences, Beijing 100049, China
* Correspondence: shangjie@nimte.ac.cn (J.S.); runweili@nimte.ac.cn (R.-W.L.)
† These authors contributed equally to this work.

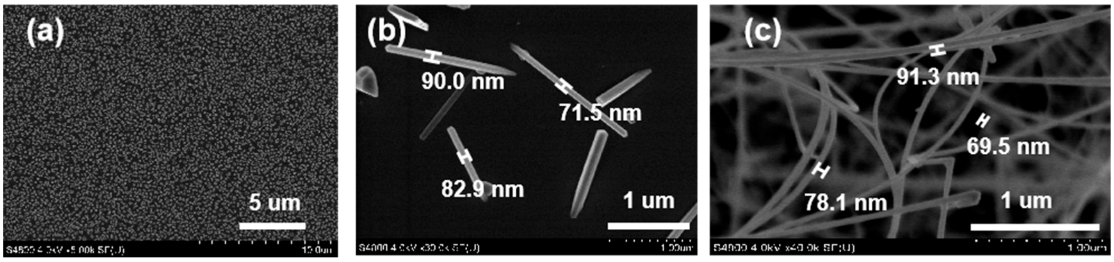

Figure S1. Preparation of Au nanoparticles and the effect of the catalyst on the morphology of Ga_2O_3 nanowires grown by CVD.

(a) SEM image of Au nanoparticles after high-temperature annealing.

(b) SEM image of Ga_2O_3 nanowires grown on an ultrathin silicon substrate by CVD without a catalyst.

(c) SEM image of Ga_2O_3 nanowires grown on an ultrathin silicon substrate by CVD with Au nanoparticle catalyst.

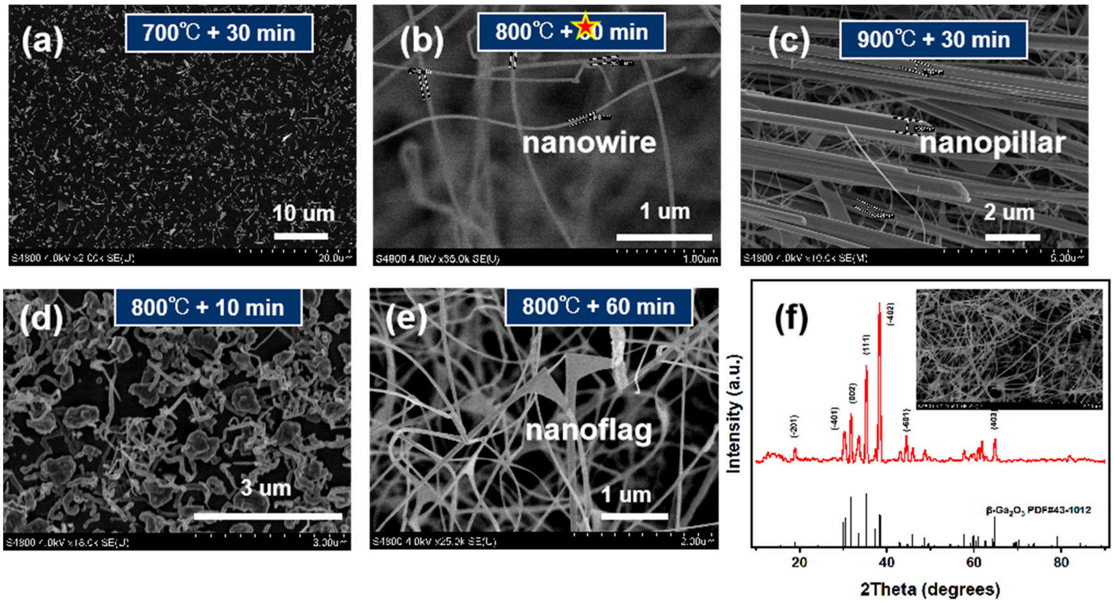

Figure S2. The effect of growth temperature and time on the morphology of Ga_2O_3 nanowires prepared by catalyst-assisted CVD method. The growth temperature and time are as follows: (a) 700°C, 30 min; (b) 800°C, 30 min; (c) 900°C, 30 min; (d) 800°C, 10 min; (e) 800°C, 60 min, showing the microscopic morphology of Ga_2O_3 nanowires. (f) XRD characterization of Ga_2O_3 nanowires grown at 800°C for 30 min, with the inset showing the SEM image of Ga_2O_3 nanowires under this growth condition.

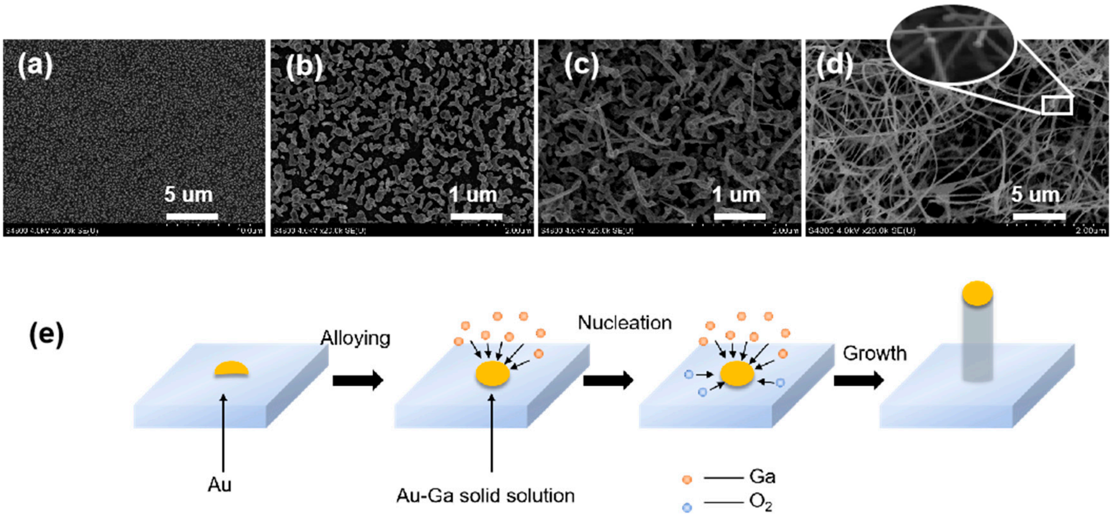

Figure S3. Growth mechanism of Ga_2O_3 nanowires prepared by CVD method on ultrathin silicon substrates. The growth temperature and time are as follows: (a) 800°C, 0 min; (b) 800°C, 10 min; (c) 800°C, 20 min; (d) 800°C, 30 min, showing the microscopic morphology of Ga_2O_3 growth; (e) Schematic diagram of the VLS growth mechanism of Ga_2O_3 nanowires.

The growth process mainly includes: Au nanoparticle catalysts are uniformly dispersed on the substrate (Figure S3(a)), utilizing Au's excellent atomic adsorption ability, the precursor Ga condenses on the gold surface, forming Au-Ga solid solution [Reference 41 in the main text]. As Ga precursor in the gas phase continues to supply, in the presence of oxygen, the solubility of Ga and oxygen atoms in the liquid droplet reaches saturation, and Ga_2O_3 crystallization starts (Figure S3(b)), gradually forming the solid structure of nanowires (Figure S3(c)). At the end of growth, spherical Au particles are visible at the tips of the nanowires, verifying the VLS growth mechanism (Figure S3(d)). Therefore, the Au nanoparticle catalyst promotes the axial growth of the nanowires, increases the aspect ratio, and makes the nanowires interwoven and entangled, which is beneficial for the fabrication of flexible daylight-blind ultraviolet detectors. Under optimal conditions, the schematic diagram of the VLS mechanism nanowire growth process is shown in Figure S3(e), which includes three steps: alloying, nucleation, and growth [Reference 40 in the main text].

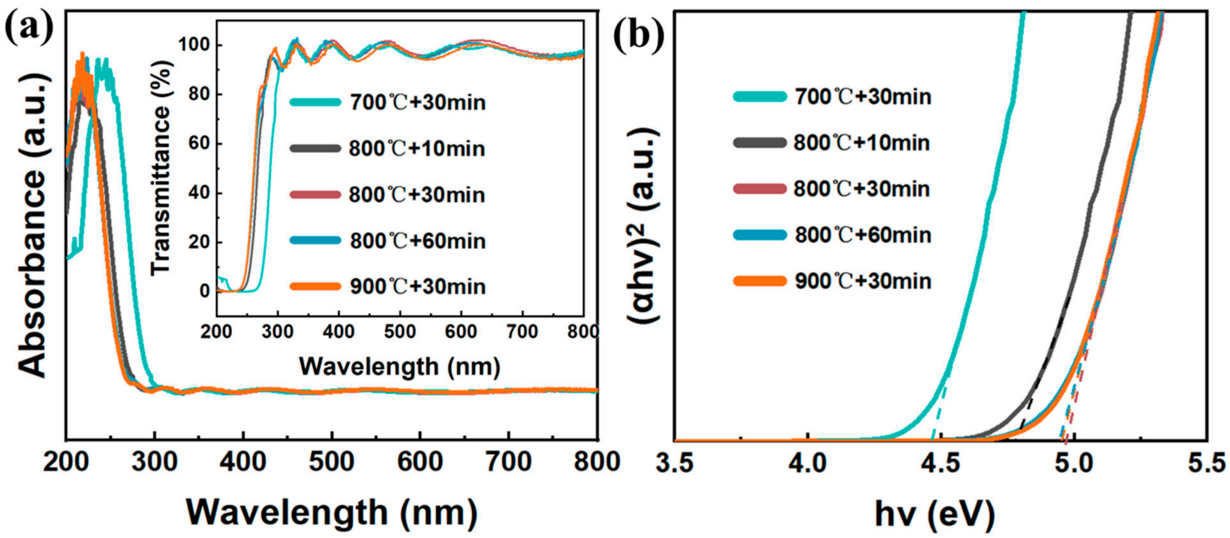

Figure S4. The effect of different growth parameters on the daylight-blind ultraviolet absorption and optical bandgap of Ga_2O_3 nanowires prepared by catalyst-assisted CVD method.
(a) Absorption and transmission spectra of Ga_2O_3 nanowires with different growth parameters.
(b) Estimation of the optical bandgap.

Sample	Growth temperature (°C)	Growth time (min)	Band gap (eV)
1	700	30	≈4.46
2	800	10	≈4.79
3	800	30	≈4.96
4	800	60	≈4.94
5	900	30	≈4.95

Table S1. Estimation results of the optical bandgap of Ga_2O_3 nanowires prepared by catalyst-assisted CVD method with different growth parameters.

From the above results, we can observe slight variations in the bandgap under different conditions. For example, at (800° C + 30 minutes), (800° C + 60 minutes), and (900° C + 30 minutes), the bandgap values are 4.96, 4.94, and 4.95 eV, respectively. These variations may indicate a potential influence of temperature and time on the bandgap, although the changes are minimal and could be affected by experimental conditions and measurement tolerances, making this effect not particularly significant. However, the precise impact of the temperature/time effect requires further in-depth investigation.

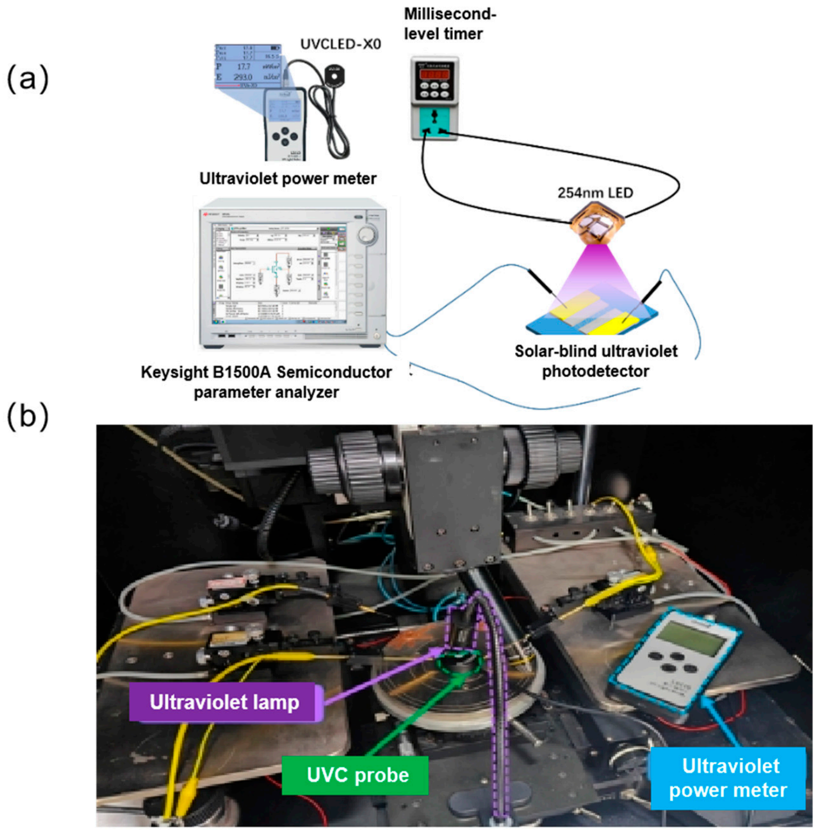

Figure S5. (a) Schematic diagram and (b) photograph of the optoelectronic testing system. The self-built optoelectronic testing system mainly includes the B1500A semiconductor parameter analyzer, 254 nm daylight-blind ultraviolet light source, ultraviolet power meter, UVC probe, and timer.

Photoelectric Testing Process

The light source used is a 254 nm and 365 nm light source from Zhongshan Zigu Lighting, with the light power density varying non-linearly from 13 mW cm^{-2} to 0.1 mW cm^{-2}as the irradiation distance changes from 0 to 90 mm. The spot diameter ranges from 18 mm to 85 mm. The ultraviolet power meter is an LS125 model from Linshang Technology, used with a UVCLED-X0 probe to measure the light power density of the ultraviolet light at specific distances. This ultraviolet power meter can display power, energy, and maximum and minimum power values in real-time. The probe has a spectral response range of 230-280 nm, which meets the requirements for the blind ultraviolet band, with a measurement range of 0-200 mW cm^{-2} and a measurement aperture of 10 mm, satisfying both the light power density and spot diameter requirements for the ultraviolet light. To achieve periodic switching of the ultraviolet light, a cycle time controller from Xunni Electronics is used, with a time control accuracy at the millisecond level.

In the specific experiment, the I-V and I-T testing processes are as follows: First, the air pump and B1500A semiconductor parameter analyzer are turned on, and the device under test is fixed on the test platform. With the help of a magnifying glass, the probe is gradually brought into contact with the device. After setting the test type in the software, the corresponding equipment is configured for testing. The I-V test requires the selection of the scanning voltage range and step

size. In this study, the voltage range is from -20 V to 20 V, with a step size of 0.02 V. When ultraviolet light irradiation is required, the probe is first lifted, the device is removed, the LED light of the required wavelength is turned on, and the ultraviolet power meter is used to measure the power, adjusting the irradiation distance to achieve the desired light power density. The device is then fixed back onto the test platform, and the probe makes contact with the device electrode again. Since there is a one-to-one correspondence between light power density and irradiation distance, we have summarized the irradiation distances corresponding to different light power densities (100 μW cm^{-2}, 200 μW cm^{-2}, 400 μW cm^{-2}, 800 μW cm^{-2}, and 1600 μW cm^{-2}).

The I-T test requires a fixed switching cycle for the ultraviolet light, which is achieved by connecting a timer in series with the ultraviolet LED light. To ensure that the response current reaches its maximum value, we avoid switching the ultraviolet light too quickly. In the experiment, the turning on and off times of the ultraviolet LED light are set to 5 seconds.

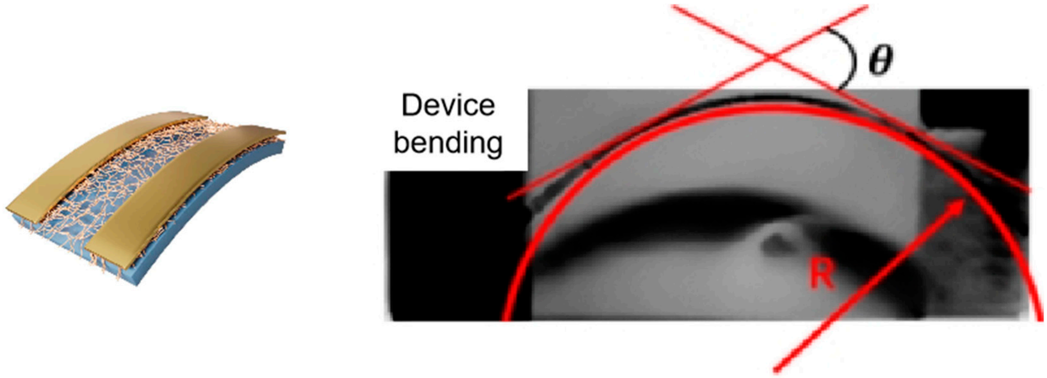

Figure S6. Schematic diagram of the bending of β-Ga_2O_3 nanowire flexible daylight-blind ultraviolet photodetector.

We use bending angles of 15°, 30°, 45°, and 60° to quantify strain, relating each angle to the bending radius r via Eq. (S1):

$$\mathrm{r} = \frac{1}{\theta \times \frac{\pi}{180^\circ}} \tag{S1}$$

where *r* is the radius of curvature, *l* is the arc length (device length) and θ is the bending angle in radians.

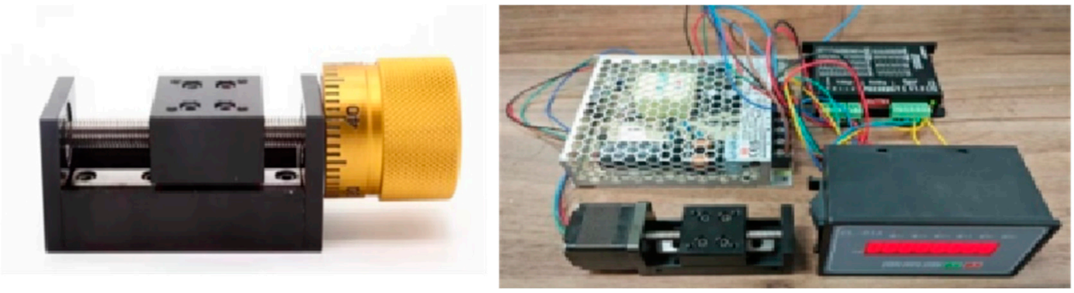

Figure S7. Strain application and strain cycling system. It mainly includes a precision linear T-type slide module with a micro handwheel, and the strain cycling system consists of a micro electric precision linear T-type slide, power supply, driver, and programmable controller.
